# Supplementary material for: Uncertainty–guided learning with scaled prediction errors in the basal ganglia
Source: PLoS Comput Biol. 2022 May 27;18(5):e1009816. doi: 10.1371/journal.pcbi.1009816 (PMC9182698; doi:10.1371/journal.pcbi.1009816)
Supplement: S1 Appendix — Fig A in S1 Appendix. The mode–matching method. (DOCX) [file pcbi.1009816.s001.docx]

# Supporting information

S1 Appendix. Derivation from Bayesian learning

One way to derive the scaled prediction error learning rules is the Bayesian mode-matching method, which is a novel (as far as we know) method to approximate Bayesian learning. We first introduce this method. We then apply the method to the problem of tracking the mean and standard deviation of a signal and thus find a new set of learning rules.

The mode-matching method

The mode-matching method is based on Bayesian principles. Let us consider the problem of learning the mean and standard deviation of a signal. A fully Bayesian learner would always maintain a belief about the values of the mean and standard deviation, encoded as a probability distribution over all possible pairs of values. It would also maintain a generative model of the signal. When the learner is provided with new information (say another sample of the signal), it applies Bayes’ law to combine its current belief (now the prior) and the likelihood of the observation (computed using the generative model) into a posterior distribution, which encodes its belief after observing the sample. This process is then repeated ad infinitum, with the posterior after one sample turning into the prior for the next.

Now, consider a learner that cannot encode arbitrary belief distributions. Instead, it can only adapt a few of the parameters of a belief distribution with otherwise fixed shape. For example, it might encode a belief using a normal distribution with fixed width and update it by adapting the mean. How might such a learner—let us call it a fixed-shape learner—approximate a fully Bayesian learner best?

Here, we propose the mode-matching method: after observing a new sample, the fixed-shape learner should change the parameters of its belief distribution such that the maximum of the distribution (its mode) is aligned with the maximum of the true posterior. We show this process schematically in Fig A.


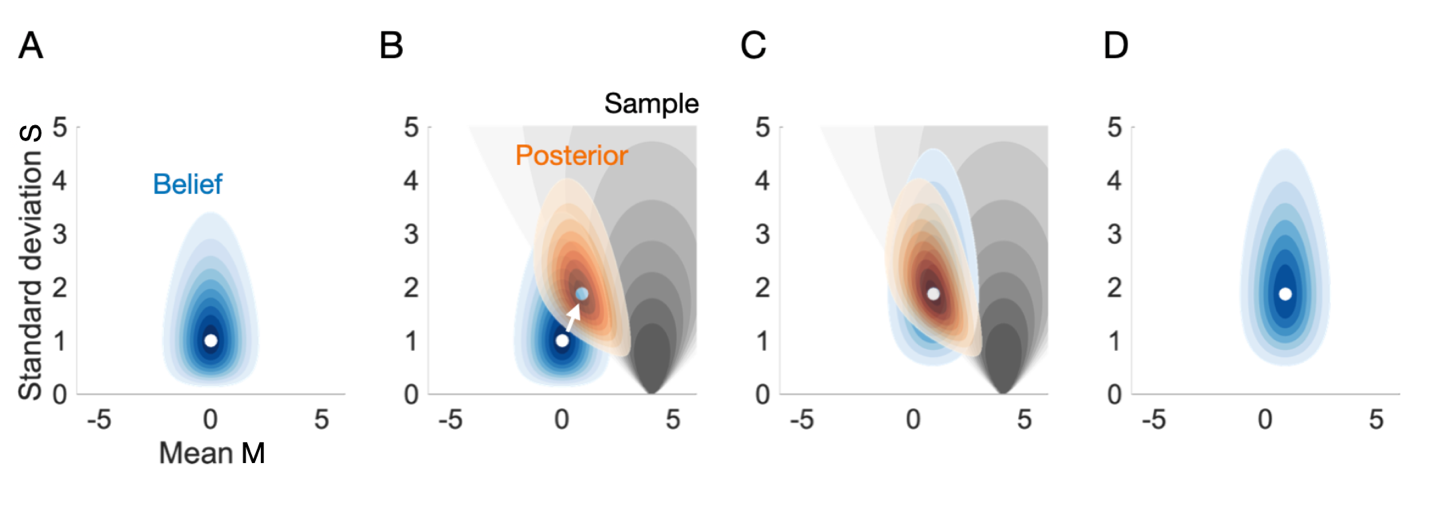


**Fig A. The mode-matching method.** The fixed-shape belief distribution is represented by a blue shading; darker shades of blue indicate higher probabilities. Similarly, the true posterior distribution is represented as an orange shading, and the likelihood of the sample is represented by a grey shading. The axes represent the mean and standard deviation of a signal. Units are arbitrary. **A** The fixed-shape belief distribution encodes the learner's knowledge before a new observation is made. **B** A new observation is made. The likelihood of the observed value is indicated by the grey shading. Using the fixed-shape belief as a prior, a posterior can be computed. The posterior's mode is different from the mode of the fixed-shape belief; therefore, an update (indicated by a white arrow) is required. **C** The fixed-shape belief has been modified such that its mode aligns with the mode of the true posterior. **D** The modified fixed-shape belief represents the learner's knowledge after the new observation has been taken into account. The distributions were computed using the densities given in Eq. S7 – S9.

After the update, the fixed-shape learner's belief is still different from the true posterior. This is because the shape of the true posterior is generally not the same as the fixed belief shape that the learner uses. Hence, mode-matching is only an approximation of Bayesian learning, and some features are lost in this approximation.

Mode-matching is formally related to the variational Bayes scheme (1-4), which works by minimizing the Kullback-Leibler divergence between the true posterior and a fixed belief shape (usually a multivariate normal distribution). However, mode-matching does not minimise the Kullback-Leibler divergence; instead, it minimises the distance between the modes of the distributions.

The learning rules that can be derived with the mode-matching method are not as precise as those derived from variational Bayes, let alone fully Bayesian learning. What makes mode-matching interesting is that it can be used to derive relatively simple, tractable learning rules, as we shall see in the next section.

New learning rules via mode-matching

Let us consider a situation in which an organism tracks the size of a reward associated with some behavior. By engaging in that behavior, it samples the reward size $r$. Using these samples, it attempts to estimate the mean reward $\mu$ that can be expected from performing the behavior at any given time.

To derive the learning rules for this situation, we start with a generative model for the reward process, and the learner's fixed-shape belief distributions over the process variables. We model rewards as normally distributed around a mean $\mu$, with a standard deviation $\sigma$, as defined in Eq. 1. Note that $\sigma$ quantifies trial-by-trial fluctuations, and therefore observation noise. The distribution in Eq. 1 is stationary; this means that the environment is modelled as stable.

We further assume that the learner maintains beliefs $M$ and $S$ about $\mu$ and $\sigma$, in form of a normal distribution over possible values of $\mu$ and a gamma distribution over possible value of $\sigma$:

$$M\sim N\left( m,\sigma_{m} \right)$$

Eq. S1

$$S\sim\Gamma(a, b)$$

Eq. S2

The learner can change its beliefs by adapting the mean (and hence mode) $m$ of the normal distribution, and the mode $\frac{a-1}{b}$ of the gamma distribution. The standard deviation $\sigma_{m}$ and the rate parameter $b$ stay fixed.

How should we interpret this belief encoding? Allowing $m$ and $s$ to vary implies that the learner considers both the mean reward $\mu$ and the observation noise $\sigma$ as unknown—it can adapt its beliefs about these variables. Fixing $\sigma_{m}$ and $b$ implies that the learner's uncertainty about the mean and the standard deviation of the signal are kept constant—it cannot adapt those. The learner will thus not become more certain about either the mean or the standard deviation as it gathers more and more data. Fixing $\sigma_{m}$ and $b$ keeps the resulting learning rules simple. An additional advantage of this design arises when the environment fails to be stationary—then, high certainty about the tracked variables would prevent the learner from adapting to new situations. The model of the reward generating process and the learner's belief system form our central assumptions—the rest follows. The learner we are about to derive will interpret all rewards it sees as being sampled from a normal distribution with fixed mean and variance, and it will make its inferences accordingly.

Now, let us use mode-matching to derive learning rules from our assumptions. To find out how the learner should update $m$ and $s$ after sampling a reward $r$, we must first find the mode of the true posterior distribution. For this, we can use a well-known way to simplify calculations. Bayes' theorem states that

$$P\left( x | \theta\right)=P(x|\theta)P(\theta)/P(x)$$

Eq. S3

with $\theta$ the parameters that are to be inferred and $x$ the data that is observed. Now we notice that

$$\log P(\theta|x)=\log P\left( x | \theta\right)+\log P(\theta)-\log P(x)$$

Eq. S4

with the last term independent of the parameters $\theta$. We can define the function

$$E=\log P\left( x | \theta\right)+\log P(\theta)$$

Eq. S5

and it is easy to see that the parameters $\theta_{\max}$ that maximize the function $E$ also maximise the posterior distribution $P(\theta|x)$ (this is true because the logarithm is strictly monotonic). The function $E$, often called ***energy*** in analogy to statistical physics, is related to the famous of free energy function which plays a key role in many contemporary theories of brain function (5-7). In the case at hand, the function $E$ is given as

$$E=\log\left( P\left( r | M,S \right) \right)+\log\left( P\left( M | m,\sigma_{m} \right)P\left( S | a,b \right) \right)$$

$$=\log\left( S^{-1}\exp\left( -\frac{1}{2}\frac{\left( r-M \right)^{2}}{S^{2}} \right)\exp\left( -\frac{1}{2}\frac{\left( M-m \right)^{2}}{\sigma_{m}^{2}} \right)S^{a-1}\exp\left( -Sb \right) \right)+C$$

Eq. S6

with $C$ a term that does not depend on $M$ or $S$, and

$$P\left( r | M,S \right)=\left( 2\pi S^{2} \right)^{-\frac{1}{2}}\exp\left( -\frac{1}{2}\frac{\left( r-M \right)^{2}}{S^{2}} \right)$$

Eq. S7

$$P\left( M | m,\sigma_{m} \right)=\left( 2\pi\sigma_{m}^{2} \right)^{-\frac{1}{2}}\exp\left( -\frac{1}{2}\frac{\left( M-m \right)^{2}}{\sigma_{m}^{2}} \right)$$

Eq. S8

$$P\left( S | a,b \right)=\frac{b^{a}}{\Gamma\left( a \right)}S^{a-1}\exp\left( -Sb \right)$$

Eq. S9

the probability density functions associated with the distributions Eq. 1, Eq. S1 and Eq. S2. To find the maximum of $E$ with respect to $M$ and $S$, and hence the mode of the posterior, we can investigate the gradient $\left( \frac{\partial E}{\partial M},\frac{\partial E}{\partial S} \right)$ of $E$, which vanishes at the maximum. Evaluation the conditions $\frac{\partial E}{\partial M}=0$ and $\frac{\partial E}{\partial S}=0$, we find

$$M_{max}-m=\frac{\sigma_{m}^{2}}{S_{max}^{2}}\left( r-M_{max} \right)$$

Eq. S10

$$S_{max}-s=\frac{1}{b}\left( \left( \frac{r-M_{max}}{S_{max}} \right)^{2}-1 \right)$$

Eq. S11

for the location $\left( M_{max},S_{max} \right)$ of the maximum of E. In Eq. S11, $s=\frac{a-1}{b}$ is the mode of the gamma distribution.

To interpret these equations, note that the left-hand side yields the distance of $S_{max}$ and $M_{max}$ from the mode of their respective prior distributions. The right-hand side quantifies the mismatch between what was expected based on $M_{max}$ and $S_{max}$ and what actually happened: based on $M_{max}$ and $S_{max}$, the reward $r$ was expected to be close to $M_{max}$ and $\left( r-M_{max} \right)^{2}$ was expected to be close to ${S_{max}}^{2}$. The mismatches are weighted with a measure of prior narrowness, $\sigma_{m}^{2}$ in Eq. S10 and $\frac{1}{b}$ in Eq. S11.

We now must solve these equations for $M_{max}$ and $S_{max}$ to find the mode of the true posterior. We could try and find the exact solutions, but considering that the equations are nonlinear, we would have to expect complicated expressions. Here we will not choose that route: we shall restrict ourselves to approximate solutions.

We focus on the scenario in which the priors of both $M_{max}$ and $S_{max}$ are very narrow. Formally, this corresponds to $\sigma_{m}^{2}\ll1$ and $\frac{1}{b}\ll1$, or equivalently $\sigma_{m}^{2}\sim\epsilon$ and $\frac{1}{b}\sim\epsilon$ with $\epsilon\ll1$. To derive an approximate solution for this regime, we use expansions

$$M_{max}=M_{max, 0}+M_{max,1}+O(2)$$

Eq. S12

$$S_{max}=S_{max, 0}+S_{max,1}+O(2)$$

Eq. S13

for the variables we want to solve for. Here, $M_{max,1}\sim\epsilon$ and $S_{max,1}\sim\epsilon$ are first order terms with respect to the small constants $\sigma_{m}^{2}$ and $\frac{1}{b}$. These expansions can be thought of as Taylor expansions of the variables of interest, keeping only terms up to first order. To determine the zeroth and first order terms, we insert these expansions into Eq. S10 and Eq. S11 and collect all terms of a certain order. Using this procedure, we obtain

$$M_{max,0}=m$$

Eq. S14

$$S_{max,0}=s$$

Eq. S15

for the zeroth order and

$$M_{max,1}=\frac{\sigma_{m}^{2}}{S_{max,0}^{2}}\left( r-M_{max,0} \right)=\frac{\sigma_{m}^{2}}{s^{2}}\left( r-m \right)$$

Eq. S16

$$S_{max,1}=\frac{1}{b}\left( \left( \frac{r-M_{max,0}}{S_{max,0}} \right)^{2}-1 \right)=\frac{1}{b}\left( \left( \frac{r-m}{s} \right)^{2}-1 \right)$$

Eq. S17

for the first order, where the zeroth order results in Eq. S14 and Eq. S15 were already used. Reinserting these contributions into Eq. S12 and Eq. S13, we find that the mode of the posterior is approximately at

$$M_{max}=m+\frac{\sigma_{m}^{2}}{s^{2}}\left( r-m \right)+O(2)$$

Eq. S18

$$S_{max}=s+\frac{1}{b}\left( \left( \frac{r-m}{s} \right)^{2}-1 \right)+O(2)$$

Eq. S19

where $O(2)$ reminds us that we have neglected terms of second or higher order in $\frac{1}{b}$ and $\sigma_{m}^{2}$. The mode of the posterior is now found—at least approximately. The final step of the mode-matching method consists in updating the mode of the fixed-shape belief distribution—which is $(m, s)$—by aligning it with the maximum of the true posterior, which is (approximately) given by $(M_{max}, S_{max})$ in Eq. S18 and Eq. S19.

If we were just looking for computationally lightweight learning rules that approximate Bayesian learning, we could stop here. However, we are ultimately interested in modelling learning in biological systems, in particular the basal ganglia system. We must hence consider that changes in synaptic strength can only depend on local information (such as pre- and postsynaptic potentials) and low-dimensional global feedback signals (such as dopamine release in the striatum). We can achieve this here by applying yet another set of approximations. First, we identify certain factors as learning rates: $\frac{\sigma_{m}^{2}}{s^{2}}$ is replaced by $\alpha_{m}$, and $\frac{1}{b}$ by $\alpha_{s}$. Then, we simplify the equations by making the learning rates constant; we hence omit the $s$-dependence of $\alpha_{m}$. With these changes, we arrive at the learning rules specified in Eq. 2 – 4. These rules feature a global feedback signal $\delta$ and track the mean reward $m$ as well as the observation noise $s$. Both $m$ and $s$ are fed back into the learning system as they enter what we will call the ***scaled*** prediction error $\delta$.

References

1. Dayan P, Hinton GE, Neal RM, Zemel RS. The helmholtz machine. Neural computation. 1995;7(5):889-904.

2. Kingma DP, Welling M. Auto-encoding variational bayes. arXiv preprint arXiv:13126114. 2013.

3. Bogacz R. A tutorial on the free-energy framework for modelling perception and learning. Journal of mathematical psychology. 2017;76:198-211.

4. Buckley CL, Kim CS, McGregor S, Seth AK. The free energy principle for action and perception: A mathematical review. Journal of Mathematical Psychology. 2017;81:55-79.

5. Friston K. The free-energy principle: a unified brain theory? Nature reviews neuroscience. 2010;11(2):127-38.

6. Bogacz R. Dopamine role in learning and action inference. Elife. 2020;9:e53262.

7. Gershman SJ. What does the free energy principle tell us about the brain? arXiv preprint arXiv:190107945. 2019.
