# Supplementary material for: Uncertainty–guided learning with scaled prediction errors in the basal ganglia
Source: PLoS Comput Biol. 2022 May 27;18(5):e1009816. doi: 10.1371/journal.pcbi.1009816 (PMC9182698; doi:10.1371/journal.pcbi.1009816)
Supplement: Attachment — Submitted filename: response.docx [file pcbi.1009816.s002.docx]

# Response to the comments of the Reviewers

We would like to thank the Reviewers for careful reading of the manuscript and very valuable comments. We feel that the suggested modifications have significantly improved the paper. Below we describe how each of the points raised has been addressed. The comments are quoted in blue italics. The parts of the manuscript that have been substantially modified are in red ink in a version of the manuscript with tracked changes.

## Reviewer #1

The authors propose in the present paper a neurocircuitry that might underlie the precision-weighting of prediction errors. The paper is well written, and their proposed model is interesting. Some sections constitute less of a novel contribution to the field though. Some rewriting and context would benefit particular sections, to clarify the novelty of the contributions.

The paper has been modified in multiple places to clarify the context and novel contributions according to specific suggestions given in detailed comments of the Reviewer.

Additionally, a sentence has been added in the Introduction explicitly stating the key new contribution of the paper (lines 84-87).

Moreover, in the abstract, we added more emphasis on the neural implementation (that is the main novel contribution of the paper) and shortened the description of model performance.

The first part of the paper discusses their scale prediction error model, and perform simulations to demonstrate the model outperforms a more simple RW-model. This section is quite expansive, particularly since there have been many models that attempt to deal with uncertainty in prediction errors, and therefore does not constitute a novel contribution to the field. That is, researchers have covered the importance of precision-weighting of prediction errors, and have proposed various computational models that could account for such processes. For example see the work of Matthew Nassar, Chris Mathys, but also Schultz’s group, who worked on the Tobler findings that are central to this paper, who have done much work on precision-weighting of prediction errors in humans and the role of dopamine herein (For example see Diederen et al., 2015, 2016; 2017; Haarsma et al., 2020). I think the paper would benefit from mentioning this work when describing their particular model, to demonstrate what is novel about it, or why it might not be of importance to get into the differences between these models.

We added a new section “Models that feature scaled prediction errors” in the Discussion that describes in detail how the models mentioned by the Reviewer relate to the proposed model.

I realise that later on in the discussion different models are discussed as well, but it’s not clear to me that their present model provides a clear advantage over other models that seem to capture the processes the authors are interested as well, like the hierarchical gaussian filter for example.

We added a paragraph discussing the relationship of our model to the hierarchical gaussian filter (lines 673-679).

If it’s the case that this section more acts as a proof of principle that precision-weighting is important, rather than coming up with a novel insight in reinforcement learning, I would suggest shortening this section and emphasise this point.

The description of results shown in Fig 2 has been shortened in several places. We also added a paragraph to the first part of the Results clarifying that its goal is to test the performance of SPE learning rules as a model of basal ganglia learning (lines 188-193).

The second section discusses how the striatum might compute precision-weighted prediction errors by largely expanding on previous work by the same group on learning reward uncertainty. I think the model is of interest and constitutes a novel contribution to the field on how these mechanisms might be implicated in striatal sub-cortical loops. Some more discussion on how their model compares to other approaches might be beneficial here as well.

A paragraph has been added discussing an alternative model for computation of reward uncertainty proposed by Dabney et al. (lines 643-652).

## Reviewer #2

The authors present a new model for learning in noisy environments. The work is mostly focused on presenting a model which is biologically plausible and authors also present a detailed neuronal mechanism of the model in the basal ganglia. I think this is a timely question and the proposed solution is interesting and novel. The paper is well-written. I’m overall positive about this paper with one major caveat that I hope authors can sufficiently address.

We thank the Reviewer for this kind evaluation.

The main issue with the paper is about its link to Kalman filtering, which is important within the paper because authors present their model as a variant of Kalman filter. Authors argue that whereas the line of Kalman filtering work in the literature posits that organisms should be able to track observation noise, there is no biologically-realistic computational model for that. But the main idea behind Kalman filtering is optimal tracking, which requires tracking both posterior mean and posterior uncertainty. The proposed model does not track posterior uncertainty. Authors seem to ignore posterior uncertainty altogether, which is technically the most important facet of Kalman filtering (when both process- and observation noise are fixed but are nonzero.)

We added a new section “Kalman filter” in the Discussion, which extensively describes the relationship of the proposed SPE model to the Kalman filter, and explains why the SPE model can achieve performance comparable to the Kalman filter without tracking the posterior uncertainty. We added a new Figure 5A which illustrates that in the Kalman filter, the posterior uncertainty only changes at the start of the learning process, and then it converges to a steady-state value. Therefore, keeping track of posterior uncertainty is only important for setting the learning rate for initial trials, and ignoring it has little effect on the following learning performance.

In fact, I believe (but I’m happy to be convinced otherwise) that their model actually recovers the posterior uncertainty but authors mistakenly label it as observation noise. That’s because they assume that zero process noise in their generative process (fixed mu in Eq 1). In Appendix S2, authors show that the model is equivalent to the Kalman filter when the ratio of observation- to process- noise is infinite. That only happens if one assumes that the process noise is zero.

We clarified that it is indeed possible to recover posterior uncertainty from variances of observation and process noise in the steady-state Kalman filter (lines 537-539). However, we explained that the uncertainty learned by the SPE model does not correspond to the posterior uncertainty, by using an example (lines 545-548), and adding new Equation 22 that makes the relationship between the uncertainties very explicit.

These are all fine for a descriptive model, but I think these assumptions ruin the link to the normative learning mechanism of Kalman filter. And the main problem is that it is not clear why we do need to make such a huge sacrifice from the normative point: does tracking posterior uncertainty really require some process that are so different from the current model?

To demonstrate that ignoring posterior uncertainty does not ruin the performance of the model, we simulated the Kalman filter in the reward tracking task and plotted its performance in Figure 2B. It shows that the SPE model achieves very similar performance to the Kalman filter (when the observation noise is of similar magnitude or larger than the process noise).

Perhaps the strongest aspect of the proposed model is the basal ganglia mechanism for implementing the model at the neuronal level. I found that part timely and novel, but again I believe the same neuronal mechanism can be told for a model that scales prediction error with posterior uncertainty in the more general case that the process noise is nonzero.

As explained above, we explicitly stated that our model does not learn posterior uncertainty (lines 545-548).

A number of minor points:

- I suggest to move the proof in the “fixed point analysis” section to Methods to enhance readability of the paper for broader audience. I think that part is quite trivial for technical readers, and quite confusing for general psychologists/neuroscientists; so in both case does not help to present it in the forefront of results section.

We have moved the fixed-point analysis to Methods as suggested, and in the Results section we added an intuition summarizing this analysis (lines 165-172).

- I found the discussion on the free energy models too vague. Maybe authors can explain more about those models and their relation to this work, especially given the recent work by Bogacz (2020).

We expanded the discussion of the differences between the scaling of prediction error in the proposed model and the free-energy models including that by Bogacz (2020) (lines 662-669).

- I also found the discussion on AI models quite speculative. I think there might be a weak connection between these models and deep RL models, but that is very non-specific (i.e. many other biological learning model might have similar connections). I think deep RL models are not competitors of the current model. Also they do not help to understand the current work.

The section discussing the relationship to other models in AI has been removed.

- In the introduction, authors seem to imply that particle filtering is not biologically realistic without providing any reason why that is so (quite in contrary, there are influential papers arguing plausibility of Monte Carlo models for biological learning, eg Courville and Daw 2006; Gershamn et al, 2010). Relatedly, it is stated in the introduction that the model by ref 9 is not suitable to describe biological learning. While it is true that the focus of that work is the computational level, but that does not mean that it is not suitable to describe biological learning on the mechanistic or algorithmic level, especially given that it is a combination of particle filtering and Kalman filtering.

The sentence to which Reviewer referred to has been rephrased as: “it is unclear how the computations in the model of Piray and Daw (9) could be implemented in biological neural circuits”.
